# Transcriptomic profiles of human livers undergoing rewarming machine perfusion before transplantation—first insights

**DOI:** 10.1007/s10142-021-00781-0

**Published:** 2021-03-17

**Authors:** Dieter Paul Hoyer, Sandra Swoboda, Juergen Walter Treckmann, Tamas Benkö, Andreas Paul, Nahal Brocke-Ahmadinejad

**Affiliations:** 1 General, Visceral and Transplantation Surgery, University Hospital Essen, Essen, Germany; 2 Institute of Biochemistry and Molecular Biology I, Medical Faculty, Heinrich Heine University Düsseldorf, Düsseldorf, Germany

**Keywords:** Next-generation sequencing, Controlled oxygenated rewarming, Clinical transplantation, Reperfusion injury, Organ preservation

## Abstract

Machine perfusion by controlled oxygenated rewarming (COR) is feasible and safe in clinical application and result in a promising outcome. This study utilizes next-generation sequencing (NGS) to investigate the transcriptome of human liver tissue undergoing COR before liver transplantation. Cold-stored livers were subjected to machine-assisted slow COR for ~120 min before transplantation. Biopsies were taken before (preCOR) and after COR (postCOR) and 1 h after reperfusion (postRep). The samples were sequenced, using RNA-seq to analyze differential transcriptional changes between the different stages and treatments of the grafts. Comparison of differential gene expression preCOR and postCOR demonstrated 10 upregulated genes. postRep 97 and 178 genes were upregulated and 7 and 13 downregulated compared to preCOR and postCOR, respectively. A shift of gene expressions by machine perfusion to the TGF-beta pathway was observed. The present study demonstrates distinct transcriptome profiles associated with machine perfusion by COR and transplantation of human livers. Such data provide a deeper understanding of the molecular mechanisms of machine perfusion technology in human liver transplantation.

**Supplementary Information:**

The online version contains supplementary material available at 10.1007/s10142-021-00781-0.

## Introduction

Key elements in liver transplantation are allograft procurement, preservation, and reperfusion in the recipient. Extensive research analyzed mechanisms whereby this cascade of anoxia and reoxygenation leads to injury of the allograft. This so-called ischemia-reperfusion injury results in parenchymal cell death, microcirculatory failure, and inflammatory immune responses. The intensity of this injury is clinically observed by the development of good graft function, early allograft dysfunction, or even primary non-function.

Thus, research in the past decades focused on the reduction of ischemia-reperfusion injury. Most relevant progress, with translation from preclinical models into clinical studies, was shown for the application of different perfusion technologies (Hoyer et al. 2016a; Guarrera et al. 2015; Minor et al. 2013; Fondevila et al. 2012; Hoyer et al. 2016b). We described in a series of preclinical and clinical studies the protective effect of incremental temperature management during machine perfusion, resulting in clinical benefit for the liver transplant recipient. However, the underlying principles are only in part understood (Hoyer et al. 2016b; Leducq et al. 1998). Cell stress, cytotoxicity, and energy depletion in liver cells have been intensively analyzed before (Gore et al. 2000; Anatskaya and Vinogradov 2010) but cannot be applied directly to the perfusion situation.

The evolving technique of next-generation sequencing opened new doors for a deeper understanding of pathophysiological processes of liver regeneration, diseases and in the field of liver transplantation (Nicastro and D’Antiga 2018; Chen et al. 2018; Lawrence et al. 2019; Yang et al. 2020). Indeed, the field of machine perfusion of liver allografts lacks the application of these new up-to-date techniques, which will yield to gain insights into the molecular principles that underlie the protective effects of allograft machine perfusion before liver transplantation. Those findings are promising to unravel the process of ischemia-reperfusion injury in liver transplantation. Especially, the impact of temperature shifts during machine perfusion with the commencement of metabolic/transcriptomic activities requires further understanding.

The present study aimed to investigate RNA expression profiles through RNA-seq before and after human liver preservation/reconditioning by COR and after reperfusion.

## Methods

### Patient population and clinical data

All COR applications were performed between March 2014 and June 2016 and utilized extended criteria donor allografts. All detailed clinical data were described previously (Hoyer et al. 2020). Five cases were selected based on tissue availability for the current investigations. Recipients gave written informed consent to transplantation with marginal allografts and to all study-related procedures.

All data were collected prospectively and obtained from electronic clinical databases, including donor and recipient demographics, transplant surgery details, and postoperative outcomes.

Cold ischemia time was defined as the time from donor cross-clamp to removal from ice to connection to the perfusion device. Warm ischemia time was defined as the time from removal from the perfusion device to portal perfusion.

### Ethical approval

The Ethics Committee of the University Hospital Essen approved the underlying study procedures (13-5409-BO).

### Machine perfusion (COR)

Detailed descriptions of the applied perfusion technology can be found elsewhere (Hoyer et al. 2016a). Briefly, upon arrival, allografts were revisited by an experienced transplant surgeon for the suitability of transplantation. COR was carried out in a standardized fashion. Hepatic artery and portal vein were cannulated and separately perfused. Pulsatile perfusion pressure was used for hepatic artery, while continuous perfusion pressure was used for the portal vein. Pressures were set at 25 mmHg (60 bpm) and 2–4 mmHg, respectively. For machine perfusion, 2 L of Custodiol-N (Dr. Köhler Chemie, Bensheim, Germany) was utilized and the solution actively oxygenated with 100% oxygen. Perfusion was started at 10 °C and was gradually increased to 12 °C, 16 °C, and 20 °C after 30, 45, and 60 min, respectively.

### Surgery and immunosuppression

Procurements were carried out in standard fashion as defined by EUROTRANSPLANT (Wunderlich et al. 2011). Transplantation was performed with caval replacement and end-to-end-anastomoses of the hepatic artery, portal vein, and bile duct. Bypass techniques are not used at our center. The regimen of immunosuppression was standardized utilizing intravenous corticosteroids intraoperatively, followed by a reduction scheme, calcineurin inhibitors (tacrolimus, trough level 6–8 ng/mL), and mycophenolate mofetil (0.5–1 g, twice daily). All patients were sent to ICU after transplantation and monitored closely.

### Liver biopsies/specimen

First liver core biopsies were obtained from each allograft immediately after removal from the ice bag and before back table preparation (preCOR). After performance of the COR machine perfusion protocol, a second core biopsy was taken before removal from the device (postCOR). A third biopsy was taken approximately 1 h after reperfusion of the allograft in the recipient, before the closure of the abdomen (postRep). Biopsies were collected in liquid nitrogen and stored at − 80 °C until further processing.

### RNA preparation and NGS

RNA isolation, cDNA library preparation with poly-A selection, and strand-specific paired-end Illumina sequencing (read length 150 bp) of liver biopsy samples were performed by Eurofins GATC Biotech GmbH (https://www.eurofinsgenomics.eu/).

### Quality control and read trimming

Quality control of the sequence reads was performed with FastQC (Andrews 2013). To exclude low-quality reads which may lead to mi-mapping, the reads were trimmed using Trimmomatic(Bolger et al. 2014) using the settings: ILLUMINACLIP:TrueSeq3-PE.fa:2.:30:10 for specifying adapters to be removed, removing leading and trailing low quality or N bases below a quality value of 3. The reads were scanned with the sliding window method of four bases and were cut if the average quality per window was below 15. Reads shorter than 50 bases were skipped.

### Counting reads per gene/transcripts

The tool Kallisto (Bray et al. 2016) was used to quantify abundances of transcripts in the reference transcriptome Homo sapiens cDNA from Ensembl (ftp://ftp.ensembl.org/pub/release-96/fasta/homo_sapiens/cdna/ ) release 96.

The abundances are stored in individual files per each sample and were summarized to a count table (Suppl. Table S1) which was used to perform differential expression analysis.

### Differentially expressed genes

The web-version of the tool Degust (Powell n.d.) was used to perform statistical testing for differential expression and visualize the results. Degust uses an implementation of voom and limma method combination to test between samples (Law et al. 2014). Genes/transcripts with FDR < 0.05 were assigned to be statistically significant up- or downregulated.

### Functional enrichment and network visualization

We used the software Cytoscape (Version 3.7.2, 2028, Oracle Corporation) with the plug-in ClueGo (Versopm 2.5.7., May 2020) (Bindea et al. 2009) for functional enrichment analysis and classification of significantly enriched functional items in WikiPathways (08.05.2020) and Gene Ontology (GO) terms (08.05.2020). A kappa coefficient was calculated related to the interaction between pathways or terms based on gene overlap. The kappa threshold was set to 0.4. A two-sided hypergeometric test was used with Bonferroni step down for correction. The cut-off for enrichment significance was *p*<0.05. Networks were created to visualize significant pathways involved in altered molecular function and biological processes in significantly upregulated genes before and after machine perfusion and 1 h after reperfusion.

### Statistical analysis

Data were shown as the mean and standard error of the mean, or the median and range, as appropriate. A *p* value < 0.05 was considered statistically significant.SPSS version 24 (IBM, Armonk, NY, USA) was used for statistical analysis.

## Results

### Donor, recipient, and transplant characteristics

Donor and recipient demographics are given in Table 1.

**Table 1:** Clinical characteristics of donors, recipients and transplantation procedures

**Donor characteristics**	
Donor gender M/F (% of male)	4/1 (80)
Donor age, median (range)	60 (50–71)
Donor BMI, mean ± SD	28.3 (±1.7)
ICU-stay, days (median and range)	3 (1–6)
Steatosis macrovesicular	5 (0–15)
Steatosis microvesicular	10 (0–70)
CIT	524 (423–870)
DRI	2.03 (1.6–2.5)
**Recipient characteristics**	
Perfusion time, min (range)	138 (93–153)
WIT	27 (20–28)
Recipient gender M/F (% of male)	3/2 (60)
Recipient age, median (range)	64 (44–65)
Recipient LabMELD score at transplantation	15 (10–23)
**Outcome**	
ICU stay, days (median and range)	4 (2–11)
Graft survival at 1 year	100%
Recipient survival at 1 year	100%
In-house/30-day mortality (%)	0

Indication for transplantation was alcoholic liver cirrhosis in 2 recipients and hepatitis B, hepatitis C and NASH in one recipient, respectively. Hepatocellular carcinoma was present in 2 recipients.

The median Donor Risk Index was 2.03 (1.6-2.5).

Posttransplant hepatocellular injury and allograft dysfunction are graphically displayed in Supplement Figure 1. Early allograft dysfunction was not observed. In-house mortality was 0%. Graft and recipient survival after 1 year was 100%, respectively.

### Transcriptional profiles before and after perfusion and reperfusion

#### Number of reads

Quality control of the raw reads revealed overall high sequencing quality. In all samples, more than 95% of all reads were eligible for further analysis after read trimming (Suppl. Table S2). Three samples showed very low library sizes (f1_postRep, m2_postRep, m3_postRep).

Three samples in condition postRep showed very low library sizes before and after read trimming which led to an outstanding level of similarity shown in the MDS plot (Supplement Figure 2). Due to these observations, the three samples (f1_postRep, m2_postRep, m3_postRep) were excluded from statistical testing. The separation of samples f3_postRep and m5_postRep in the MDS plot with the exclusion of the other replicates is now based on the samples itself and not biased by the library size (Supplement Figure 2). Even though the exclusion of the samples reduced the number of biological replicates from five to two in condition postRep, the applied statistical methods are suited to handle different number of replicates (voom).

### Identification of DEGs and GO-term enrichment analysis

**Fig. 1:**
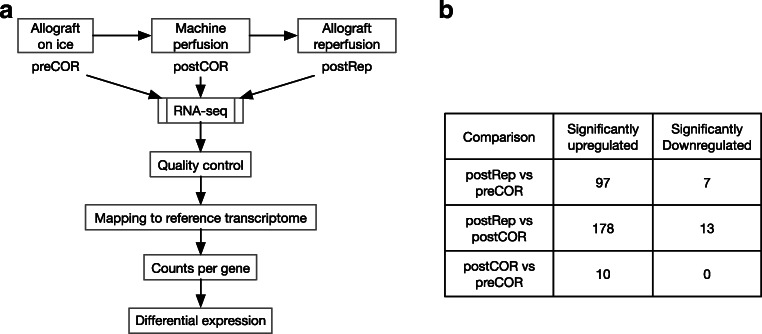
**a** Method outline. **b** Number of significantly up- and downregulated transcripts

**Fig. 2:**
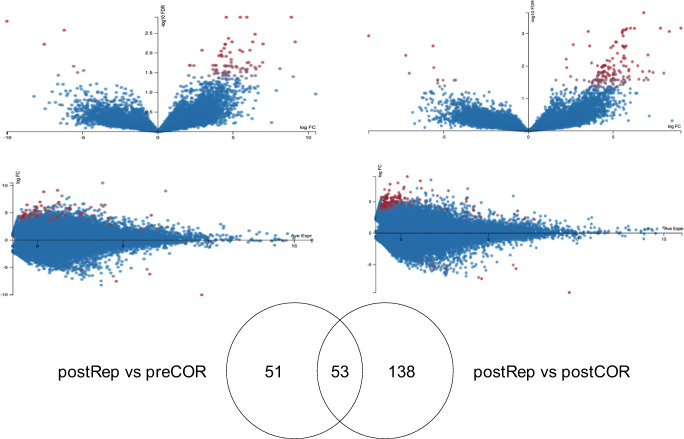
MA and Volcano plots of comparisons postRep vs preCOR and postRep vs postCOR. Overlap of significantly regulated transcripts between the two comparisons as Venn diagram

Testing of differential expression in sample *postCOR* against *preCOR* led to 10 upregulated genes (Figs. 1 and 2). Testing of sample postRep in comparison to preCOR led to 97 upregulated and 7 downregulated genes whereas comparison between postRep to postCOR yielded into 178 upregulated and 13 downregulated genes. All the differentially expressed genes (DEGs) are listed in Supplementary Table S3.

**Fig. 3:**
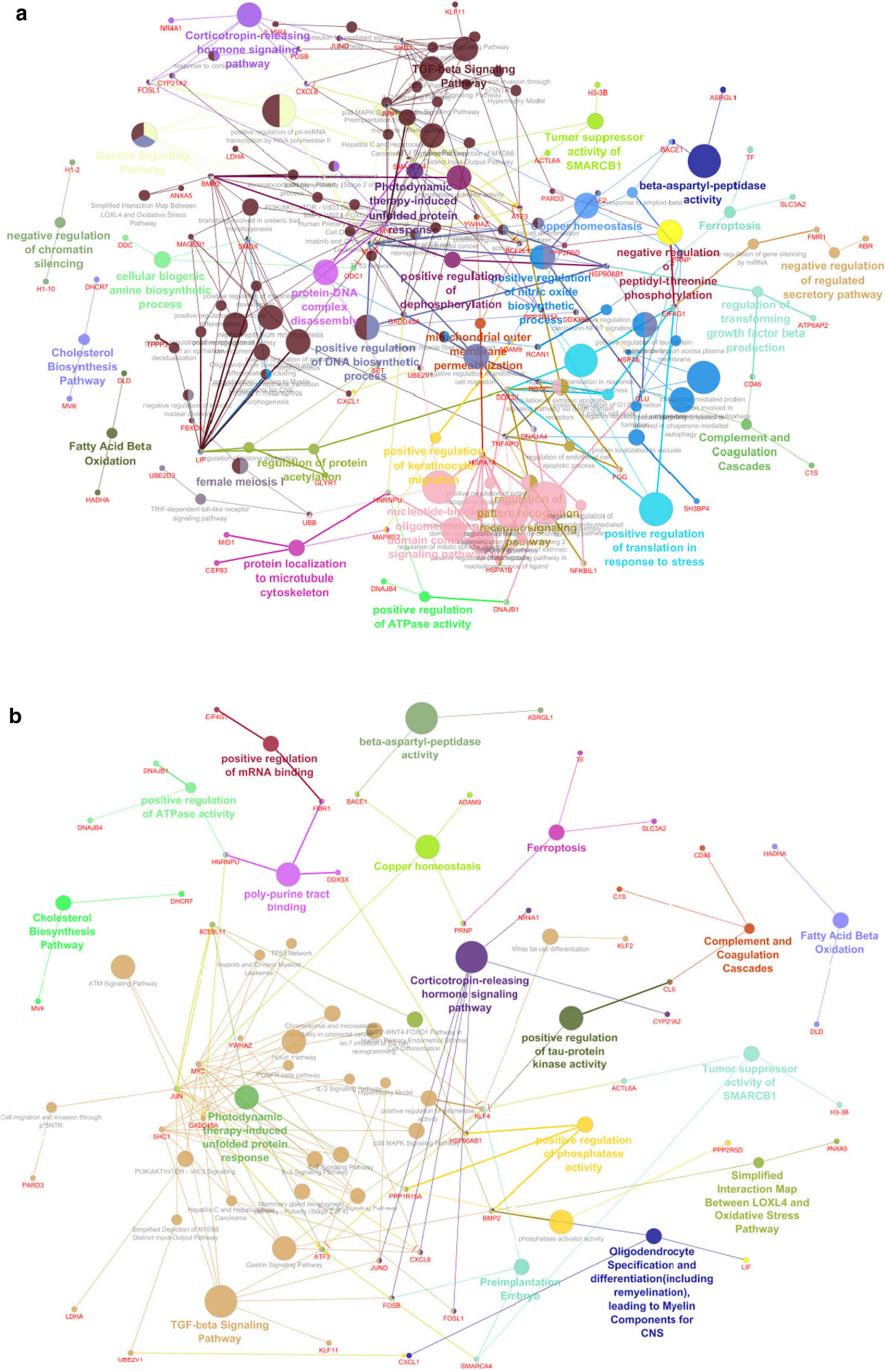
Differential expression postRep vs postCOR. **a** Network of differentially expressed biological processes. **b** Network of differentially expressed molecular functions

**Fig. 4:**
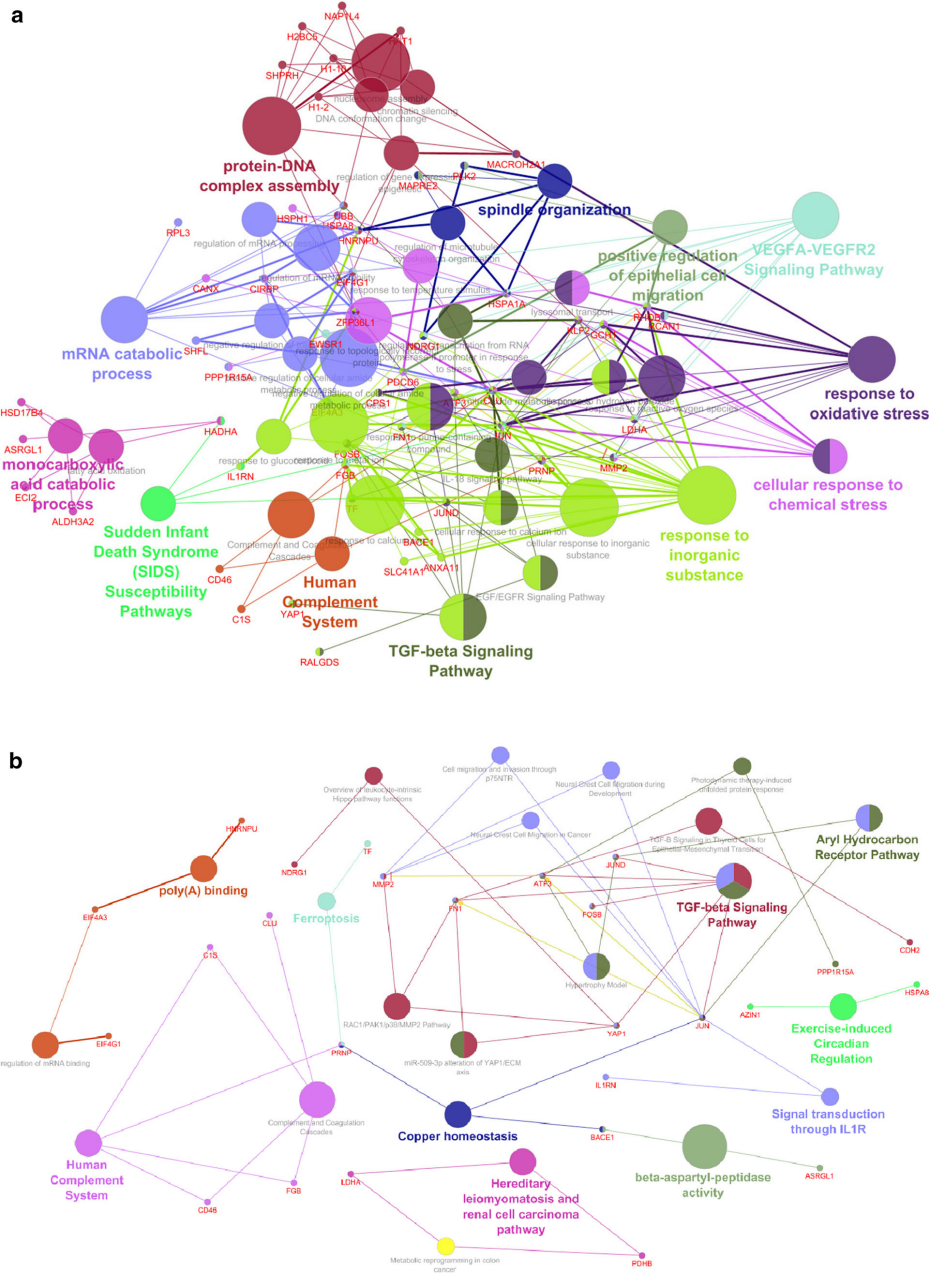
Differential expression postRep vs preCOR. **a** Network of differentially expressed biological processes. **b** Network of differentially expressed molecular functions

GO-term enrichment analysis was performed on the upregulated DEGs postRep compared to preCOR and compared to postCOR for biological processes, molecular function and cellular component. Results are depicted in detail in Figs. 3 and 4 and Supplemental Figures 3 and 4.

The upregulated genes of postRep to preCOR comparison showed 46 significantly enriched GO-terms in the category of biological process, which were grouped into 12 groups. These functions were essentially linked to ischemia-reperfusion: outstanding are responses to inorganic substances, responsive to oxidative stress, mRNA and DNA processes, and less prominent the TGF-beta signaling pathway. In the category molecular function, 27 terms in 10 groups were found related to IL1 signaling, the aryl hydrocarbon receptor pathway and less prominent again the TGF-beta signaling pathway. The third category cellular component showed 42 terms in 13 groups.

Accordingly, in the upregulated DEGs of comparison postRep to postCOR, there were 111 GO-terms found in 29 groups in the category biological process, in which the predominant pathway was the TGF-signaling pathway, besides pathways regulating nucleotide-binding oligomerization, DNA biosynthetic processes, and regulation of nitric oxide biosynthetic process.

Molecular functions demonstrated 41 terms in 18 groups. Again, the TGF-beta signaling pathway was identified as the most relevant group, followed by interactions between the LOXL4 and oxidative stress pathways. The category cellular component showed 34 terms in 16 groups.

## Discussion

This study aimed to investigate the transcriptome of human livers undergoing COR before liver transplantation before COR, after COR, and 1 h after reperfusion.

The presented data demonstrate for the first time expression profiles during the rewarming of human livers by machine perfusion. Allover, comparison of expression profiles before initiation of machine perfusion and after the rewarming period showed minimal differences on the transcriptional level, though significantly altered transcriptome profiles were identified postRep.

This indicates that livers do not alter the genome expression patterns at temperatures up to 20 °C at the given perfusion conditions. This comes not as a surprise as the metabolic activity of the organ depends on the van’t Hoff equation, meaning a decrease of the metabolic rate by 50% by reduction of the temperature by 10 °C. At 20 °C the metabolic rates of human livers should be about 30% of the normal level. Additionally, the perfusion utilizes non-physiological conditions in terms of perfusion medium, therefore electrolyte balances, glucose levels, and temperature-adapted perfusion pressures. However, the energetic demand of the organs is sufficiently met, as shown in other studies (Hoyer et al. 2016a; Minor et al. 2013). Indeed, this is the first evidence that at 20 °C the transcriptome does not change and might be relevant in potential future studies, aiming to influence the transcriptome ex vivo, before reperfusion.

These observations stand in line with other studies describing that the major changes to the liver phenotype and liver injury associated with organ procurement and preservation become apparent after reperfusion with blood in the recipient(Chu et al. 2015; Olthoff 2002). This does not preclude that interventions during preservation influence the way and degree of reperfusion injury in the recipient. First applications of COR before LT suggested a clinical benefit, recently (Hoyer et al. 2016a; Hoyer et al. 2020). Hence, the benefit of COR arises after reperfusion with blood in the recipient. The comparison of the transcriptome preCOR and postRep shows a differential upregulation of 98 genes. These included in large proportions biological processes as responses to inorganic substances, oxidative stress and mRNA, and DNA metabolism. Significant molecular functions were grouped to IL 1 signaling and the aryl hydrocarbon receptor pathway. Both analyses identified the upregulation of the TGF-beta signaling pathway to a lesser degree.

Overall, this differential gene expression is in line with other studies investigating the transcriptome after liver transplantation, describing mechanisms of ischemia-reperfusion injury and involved pathways. These studies named upfront responses to oxidative stress as well as mRNA and DNA regulations, inflammatory and apoptotic processes (Defamie et al. 2008; Confi et al. 2007).

In contrast, the transcriptome postRep compared to postCOR demonstrated 178 upregulated genes. The predominant biological processes and molecular functions were related to the TGF-beta signaling pathway followed by processes of DNA biosynthesis and regulation of nitric oxide and molecular functions of oxidative stress. This pattern of gene upregulation toward the TGF-beta pathways, when compared to the preCOR transcriptome, is of major interest as it suggests a shift of gene expressions by machine perfusion to the TGF-beta pathway.

Transforming growth factor-beta (TGF-beta) is a growth factor with multiple biological properties including stimulation and inhibition of cell proliferation and profibrogenic and anti-proliferative pleiotropic functions depending on the cellular context (Fabregat et al. 2016; Karkampouna et al. 2012). TGF-beta has been described as a key regulator in liver diseases, from initial injury through inflammation and fibrosis to cirrhosis (Dooley and Ten Dijke 2012). Moreover, the important role of TGF-beta as cytostatic and apoptotic in hepatocytes, controlling liver mass, has been pointed out (Karkampouna et al. 2012), with hyperproliferative disorders and cancer resulting from loss of TGF-beta. In the regenerating liver TGF-beta mRNA is increased up to 8-fold and peaks after hepatocyte cell division and mitosis are almost completed (Braun et al. 1988). The shift toward the TGF-beta pathway postCOR suggests advanced regeneration of the allograft by COR. Although hepatocytes respond to TGF-beta, they do not synthesize TGF-beta mRNA. Instead, liver non-parenchymal cells, particularly endothelial cells, produce TGF-beta. The upregulation observed in the study at hand can be taken as another hint that COR mediates its beneficial effect at least in part by stimulation of endothelial cells, as described before (Gallinat et al. 2013; Gallinat et al. 2012).

The sometimes controversial roles of TGF-beta, where paradoxically a growth inhibitor increases at the time of hepatocyte replication, may underlie that TGF-beta functions as an effector of an inhibitory paracrine loop. That would prevent uncontrolled hepatocyte proliferation (Braun et al. 1988). It is well known that an ongoing overexpression of TGF-beta results in liver fibrosis and cirrhosis. In the present series, all patients are well and alive without signs of liver fibrosis as documented by transient ultrasound elastography (data not shown).

Ischemia-reperfusion is inevitable in the present scenario of organ procurement, cooling and preservation, and transplantation into the warm recipient. It is of major interest to prepare the allograft for the demanding conditions during reperfusion in the recipient. COR has been proven to mediate a clinical benefit. With the present data at hand, gene expression profiles accountable for this phenomenon are suggested. Of special interest is the interaction of reactive oxygen species, which accumulate during ischemia-reperfusion, with TGF-beta (Jain et al. 2013). Further downstream in the TGF beta cascade, the cytoplasmatic signaling molecule Smad 3 has been shown to mediate ischemia reperfusion injury effects (Peng et al. 2019; Yang et al. 2018) . For now it remains speculative, but it seems reasonable that these are predominant targets for pharmacological interventions to further enhance the clinically observed effects of COR and expand the protection against ischemia-reperfusion injury provided by this preservation method. Presumably, this might in turn reduce the oxidative stress (Liu and Desai 2015) observed during ischemia reperfusion injury, which leads to inflammatory and apoptotic processes. Additionally, Smad-independent pathways (e.g., p38 MAPK, Erk pathways) are involved in cell growth regulation and cytokinesis and should be further investigated in the context of COR. Proteomic investigations might help to validate potential treatment targets.

Additionally it would be of great interest in the future to actively induce changes on a cellular level by induction of mRNA or miRNA (Budak and Zhang 2017) to the perfusion circuit to promote protective protein expressions. Thus, improvement and refinement of COR by future investigations hopefully optimizes preservation of liver allografts before transplantation, which might increase the organs’ capability of resistance against ischemia reperfusion injury and will lead to expanded preservation times.

This is the first study investigating transcriptomic profiles of COR before liver transplantation. The only other machine perfusion modality which has been investigating differential gene expressions has been normothermic machine perfusion so far (Jassem et al. 2019). By microarray investigations, a set of 12 clinically transplanted machine perfused livers were compared to livers undergoing conventional cold storage as preservation modality. Here, gene expression profiles of liver tissue were shifted from proinflammation to prohealing and regeneration by NMP.

Our work is affected by several limitations commonly described in human transplant studies: large variations in the preservation and transplantation times, caused by logistical reasons and surgical issues are present between cases; intrinsic variation of the allografts as well as the variable effect of brain death persists, which has been described by transcriptomic studies before (Colombo et al. 2006). Moreover, the small sample size without an extrinsic control group further limits the conclusions that can be drawn from these data.

In conclusion, the evolving techniques of NGS open new doors to address the partially contrary results of ischemia-reperfusion injury and liver regeneration. We delineated a differential transcriptome after liver reperfusion by COR preservation, indicating dominant pathways by which the suggested beneficial effect is potentially mediated.

## Supplementary Information

ESM 1 (PPTX 39 kb)

ESM 2 (PPTX 221 kb)

ESM 3 (PPTX 1922 kb)

ESM 4 (PPTX 1519 kb)

ESM 5 (CSV 10843 kb)

ESM 6 (DOCX 13 kb)

ESM 7 (XLSX 160 kb)

## Abbreviations

CIT: Cold ischemic time
COD: Cause of death
COR: Controlled oxygenated rewarming
DGE: Differential gene expression
DRI: Donor Risk Index
EAD: Early allograft dysfunction
F: Female
GO: Gene ontology
HA: Hepatic artery
HTK: Histidin-Tryptophane-Ketoglutarat
ICU: Intensive Care Unit
labMELD: laboratory Model for End Stage Liver Disease Score
LT: Liver transplantation
M: Male
PNF: Primary non-function
PV´: Portal vein
UW: University of Wisconsin solution
WIT: Warm ischemic time

## References

[CR1] Anatskaya OV, Vinogradov AE (2010). Somatic polyploidy promotes cell function under stress and energy depletion: evidence from tissue-specific mammal transcriptome. Funct. Integr. Genomics..

[CR2] Andrews S (2013) FastQC a quality control tool for high throughput sequence data. Available from: https://www.bioinformatics.babraham.ac.uk/projects/download.html. Accessed Feb 2019

[CR3] Bindea G, Mlecnik B, Hackl H, Charoentong P, Tosolini M, Kirilovsky A (2009). ClueGO: a Cytoscape plug-in to decipher functionally grouped gene ontology and pathway annotation networks. Bioinformatics..

[CR4] Bolger AM, Lohse M, Usadel B (2014). Trimmomatic: a flexible trimmer for Illumina sequence data. Bioinformatics..

[CR5] Braun L, Mead JE, Panzica M, Mikumo R, Bell GI, Fausto N (1988). Transforming growth factor β mRNA increases during liver regeneration: a possible paracrine mechanism of growth regulation. Proc Natl Acad Sci U S A.

[CR6] Bray NL, Pimentel H, Melsted P, Pachter L (2016). Near-optimal probabilistic RNA-seq quantification. Nat Biotechnol.

[CR7] Budak H, Zhang B (2017). MicroRNAs in model and complex organisms. Funct Integr Genomics.

[CR8] Chen X, Zhongyang S, Zhang X, Jing G, Hao Y, Cao L et al (2018) RNA sequencing reveals the suitability of cardiac death livers for transplantation. Biomed Res Int 2018:1–8 Available from: https://pubmed.ncbi.nlm.nih.gov/30519586/. Accessed Feb 2019 10.1155/2018/8217486 PMC6241343 30519586

[CR9] Chu MJJ, Vather R, Hickey AJR, Phillips ARJ, Bartlett ASJR (2015). Impact of ischaemic preconditioning on experimental steatotic livers following hepatic ischaemia-reperfusion injury: a systematic review. HPB..

[CR10] Colombo G, Gatti S, Turcatti F, Lonati C, Sordi A, Rossi G, Bonino F, Catania A (2006). Alteration in the transcriptional profile of livers from brain-dead organ donors. Transplantation..

[CR11] Confi A, Scala S, D’Agostino P, Alimenti E, Morelli D, Andria B (2007). Wide gene expression profiling of ischemia-reperfusion injury in human liver transplantation. Liver Transpl.

[CR12] Defamie V, Cursio R, Le Brigand K, Moreilhon C, Saint-Paul MC, Laurens M (2008). Gene expression profiling of human liver transplants identifies an early transcriptional signature associated with initial poor graft function. Am J Transplant.

[CR13] Dooley S, Ten Dijke P (2012). TGF-β in progression of liver disease. Cell Tissue Res.

[CR14] Fabregat I, Moreno-Càceres J, Sánchez A, Dooley S, Dewidar B, Giannelli G, ten Dijke P, the IT-LIVER Consortium (2016). TGF-β signalling and liver disease. FEBS J.

[CR15] Fondevila C, Hessheimer AJ, Maathuis M-HJ, Muñoz J, Taurá P, Calatayud D, Leuvenink H, Rimola A, García-Valdecasas JC, Ploeg RJ (2012). Hypothermic oxygenated machine perfusion in porcine donation after circulatory determination of death liver transplant. Transplantation..

[CR16] Gallinat A, Paul A, Efferz P, Lüer B, Kaiser G, Wohlschlaeger J, Treckmann J, Minor T (2012). Hypothermic reconditioning of porcine kidney grafts by short-term preimplantation machine perfusion. Transplantation..

[CR17] Gallinat A, Fox M, Lüer B, Efferz P, Paul A, Minor T (2013). Role of pulsatility in hypothermic reconditioning of porcine kidney grafts by machine perfusion after cold storage. Transplantation..

[CR18] Gore MA, Morshedi MM, Reidhaar-Olson JF (2000). Gene expression changes associated with cytotoxicity identified using cDNA arrays. Funct Integr Genomics.

[CR19] Guarrera JV, Henry SD, Samstein B, Reznik E, Musat C, Lukose TI, Ratner LE, Brown RS, Kato T, Emond JC (2015). Hypothermic machine preservation facilitates successful transplantation of “orphan” extended criteria donor livers. Am J Transplant.

[CR20] Hoyer DP, Mathé Z, Gallinat A, Canbay AC, Treckmann JW, Rauen U, Paul A, Minor T (2016). Controlled oxygenated rewarming of cold stored livers prior to transplantation: first clinical application of a new concept. Transplantation..

[CR21] Hoyer DP, Paul A, Luer S, Reis H, Efferz P, Minor T (2016). End-ischemic reconditioning of liver allografts: controlling the rewarming. Liver Transpl.

[CR22] Hoyer DP, Benkö T, Manka P, von Horn C, Treckmann JW, Paul A (2020). Long-term outcomes after controlled oxygenated rewarming of human livers before transplantation. Transplant Direct.

[CR23] Jain M, Rivera S, Monclus EA, Synenki L, Zirk A, Eisenbart J, Feghali-Bostwick C, Mutlu GM, Budinger GRS, Chandel NS (2013). Mitochondrial reactive oxygen species regulate transforming growth factor-β signaling. J Biol Chem.

[CR24] Jassem W, Xystrakis E, Ghnewa YG, Yuksel M, Pop O, Martinez-Llordella M, Jabri Y, Huang X, Lozano JJ, Quaglia A, Sanchez-Fueyo A, Coussios CC, Rela M, Friend P, Heaton N, Ma Y (2019). Normothermic machine perfusion (NMP) inhibits proinflammatory responses in the liver and promotes regeneration. Hepatology..

[CR25] Karkampouna S, ten Dijke P, Dooley S, Kruithof-de JM (2012). TGFβ signaling in liver regeneration. Curr Pharm Des.

[CR26] Law CW, Chen Y, Shi W, Smyth GK (2014). Voom: precision weights unlock linear model analysis tools for RNA-seq read counts. Genome Biol.

[CR27] Lawrence MC, Darden CM, Vasu S, Kumano K, Gu J, Wang X, Chan J, Xu Z, Lemoine BF, Nguyen P, Smitherman C, Naziruddin B, Testa G (2019). Profiling gene programs in the blood during liver regeneration in living liver donors. Liver Transpl.

[CR28] Leducq N, Delmas-Beauvieux MC, Bourdel-Marchasson I, Dufour S, Gallis JL, Canioni P (1998). Mitochondrial permeability transition during hypothermic to normothermic reperfusion in rat liver demonstrated by the protective effect of cyclosporin A. Biochem J.

[CR29] Liu RM, Desai LP (2015). Reciprocal regulation of TGF-β and reactive oxygen species: a perverse cycle for fibrosis. Redox Biol.

[CR30] Minor T, Efferz P, Fox M, Wohlschlaeger J, Lüer B (2013). Controlled oxygenated rewarming of cold stored liver grafts by thermally graduated machine perfusion prior to reperfusion. Am J Transplant.

[CR31] Nicastro E, D’Antiga L (2018). Next generation sequencing in pediatric hepatology and liver transplantation. Liver Transpl.

[CR32] Olthoff KM (2002). Molecular pathways of regeneration and repair after liver transplantation. World J Surg.

[CR33] Peng L, Yang C, Yin J, Ge M, Wang S, Zhang G, Zhang Q, Xu F, Dai Z, Xie L, Li Y, Si JQ, Ma K (2019). TGF-β2 induces Gli1 in a Smad3-dependent manner against cerebral ischemia/reperfusion injury after isoflurane post-conditioning in rats. Front Neurosci.

[CR34] Powell DR Degust: interactive RNA-seq analysis. Available from: http://degust.erc.monash.edu/. Accessed Feb 2019

[CR35] Wunderlich H, Brockmann JG, Voigt R, Rauchfuß F, Pascher A, Brose S, Binner C, Bittner H, Klar E (2011). DTG procurement guidelines in heart beating donors. Transpl Int.

[CR36] Yang T, Zhang X, Ma C, Chen Y (2018). TGF-β/smad3 pathway enhances the cardio-protection of S1R/SIPR1 in in vitro ischemia-reperfusion myocardial cell model. Exp Ther Med.

[CR37] Yang Z, Zhu H, Zhang L, Wei Q, Zhou L, Xu X, Song P, Liu J, Xie H, Zheng S (2020). DNA methylation of SOCS1/2/3 predicts hepatocellular carcinoma recurrence after liver transplantation. Mol Biol Rep.

