# Supplementary material for: Transcriptomic profiles of human livers undergoing rewarming machine perfusion before transplantation—first insights
Source: Funct Integr Genomics. 2021 Mar 17;21(3-4):367–76. doi: 10.1007/s10142-021-00781-0 (PMC8298250; doi:10.1007/s10142-021-00781-0)
Supplement: Supplementary file 1 — (PPTX 39 kb) [file 10142_2021_781_MOESM1_ESM.pptx]

## Slide 1
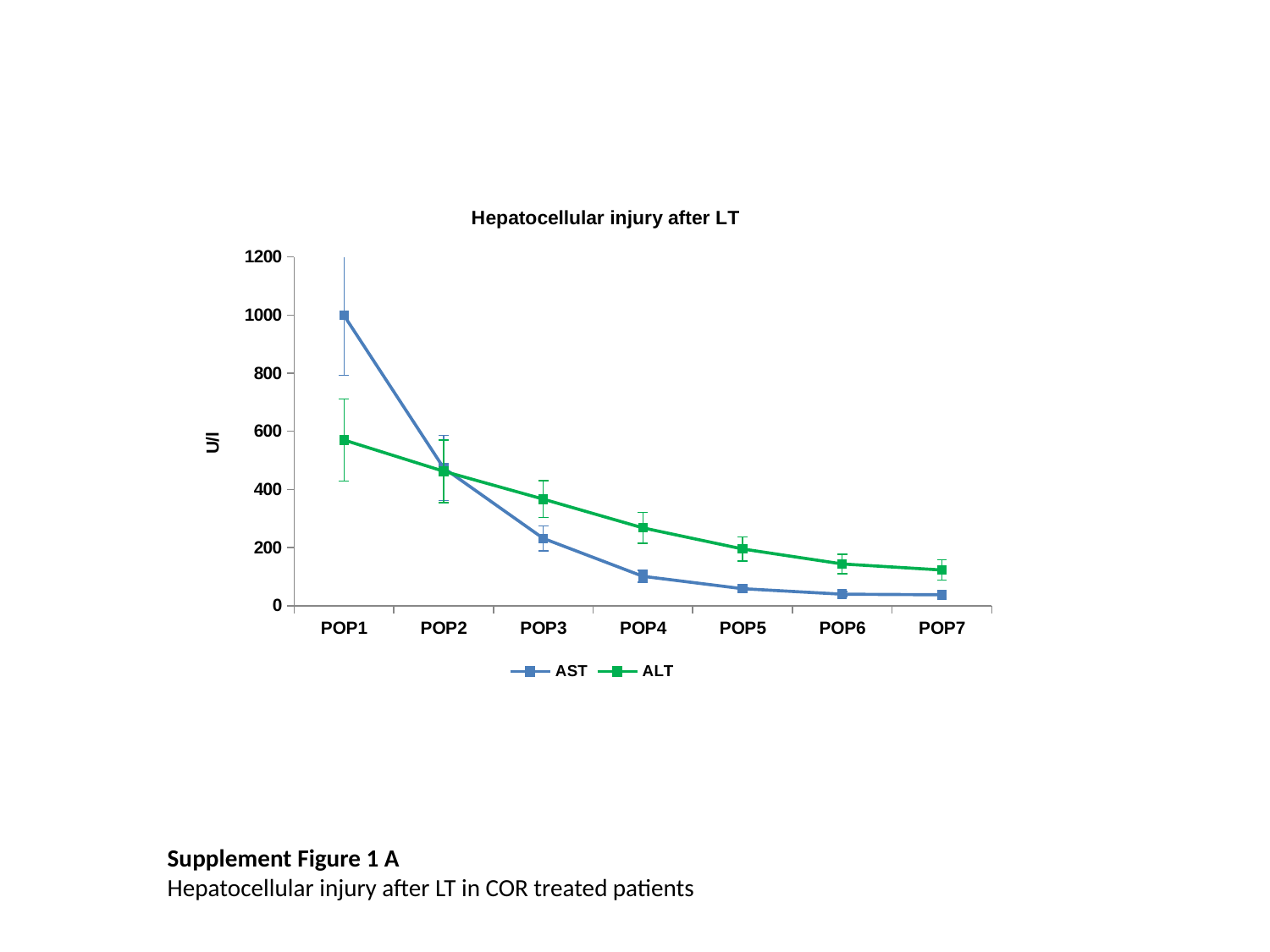

### Chart: Hepatocellular injury after LT
| Category | | |
|---|---|---|
| POP1 | 998.6 | 569.8 |
| POP2 | 473.8 | 462.4 |
| POP3 | 231.4 | 366.4 |
| POP4 | 101.0 | 267.6 |
| POP5 | 58.4 | 195.0 |
| POP6 | 39.4 | 143.4 |
| POP7 | 37.4 | 122.6 |Supplement Figure 1 A
Hepatocellular injury after LT in COR treated patients

## Slide 2
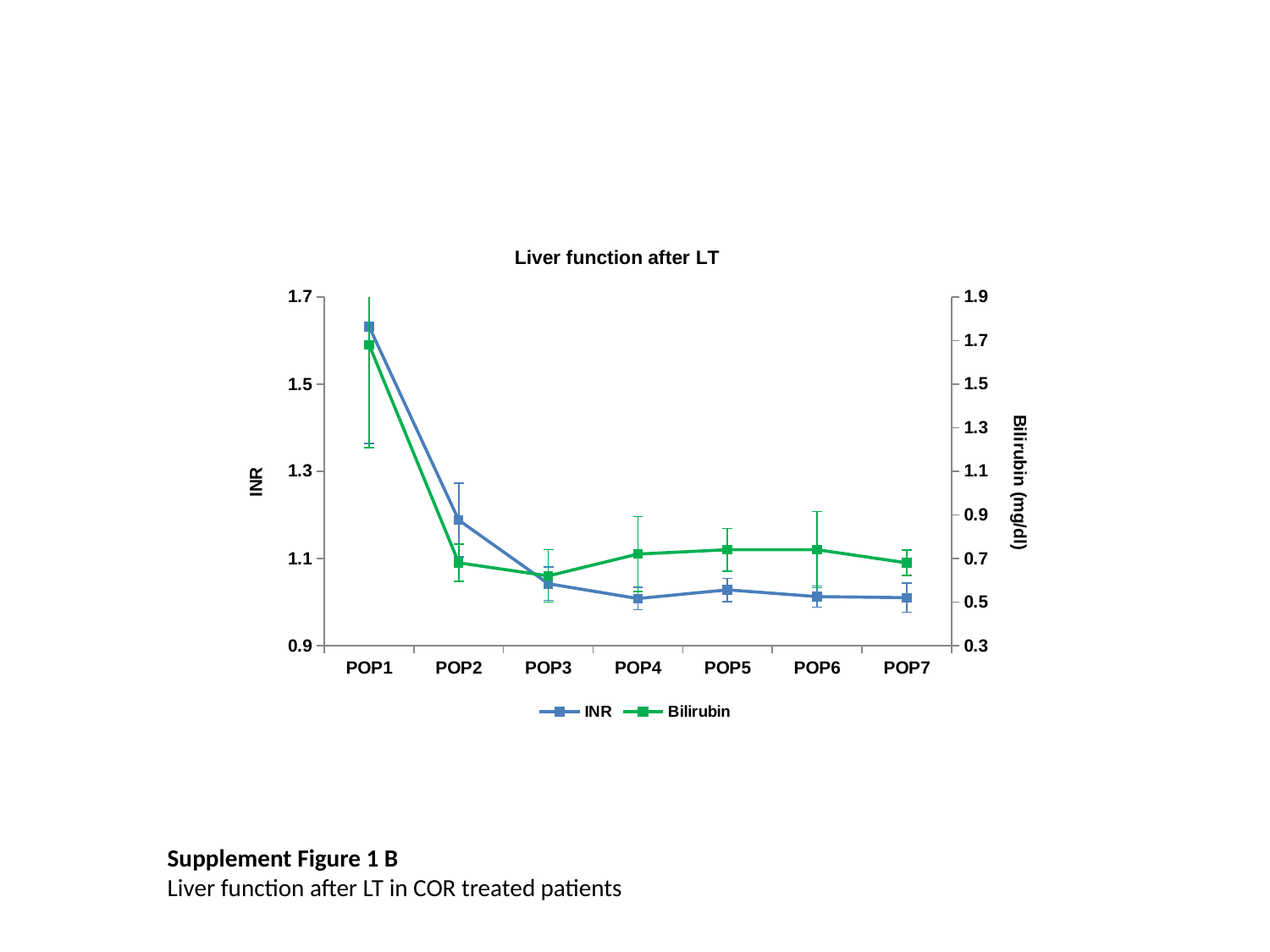

### Chart: Liver function after LT
| Category | | |
|---|---|---|
| POP1 | 1.6320000000000001 | 1.6800000000000002 |
| POP2 | 1.1880000000000002 | 0.68 |
| POP3 | 1.0419999999999998 | 0.62 |
| POP4 | 1.008 | 0.72 |
| POP5 | 1.028 | 0.74 |
| POP6 | 1.0125 | 0.74 |
| POP7 | 1.0100000000000002 | 0.68 |Supplement Figure 1 B
Liver function after LT in COR treated patients
