# Supplementary material for: Transcriptomic profiles of human livers undergoing rewarming machine perfusion before transplantation—first insights
Source: Funct Integr Genomics. 2021 Mar 17;21(3-4):367–76. doi: 10.1007/s10142-021-00781-0 (PMC8298250; doi:10.1007/s10142-021-00781-0)
Supplement: Supplementary file 2 — (PPTX 221 kb) [file 10142_2021_781_MOESM2_ESM.pptx]

## Slide 1
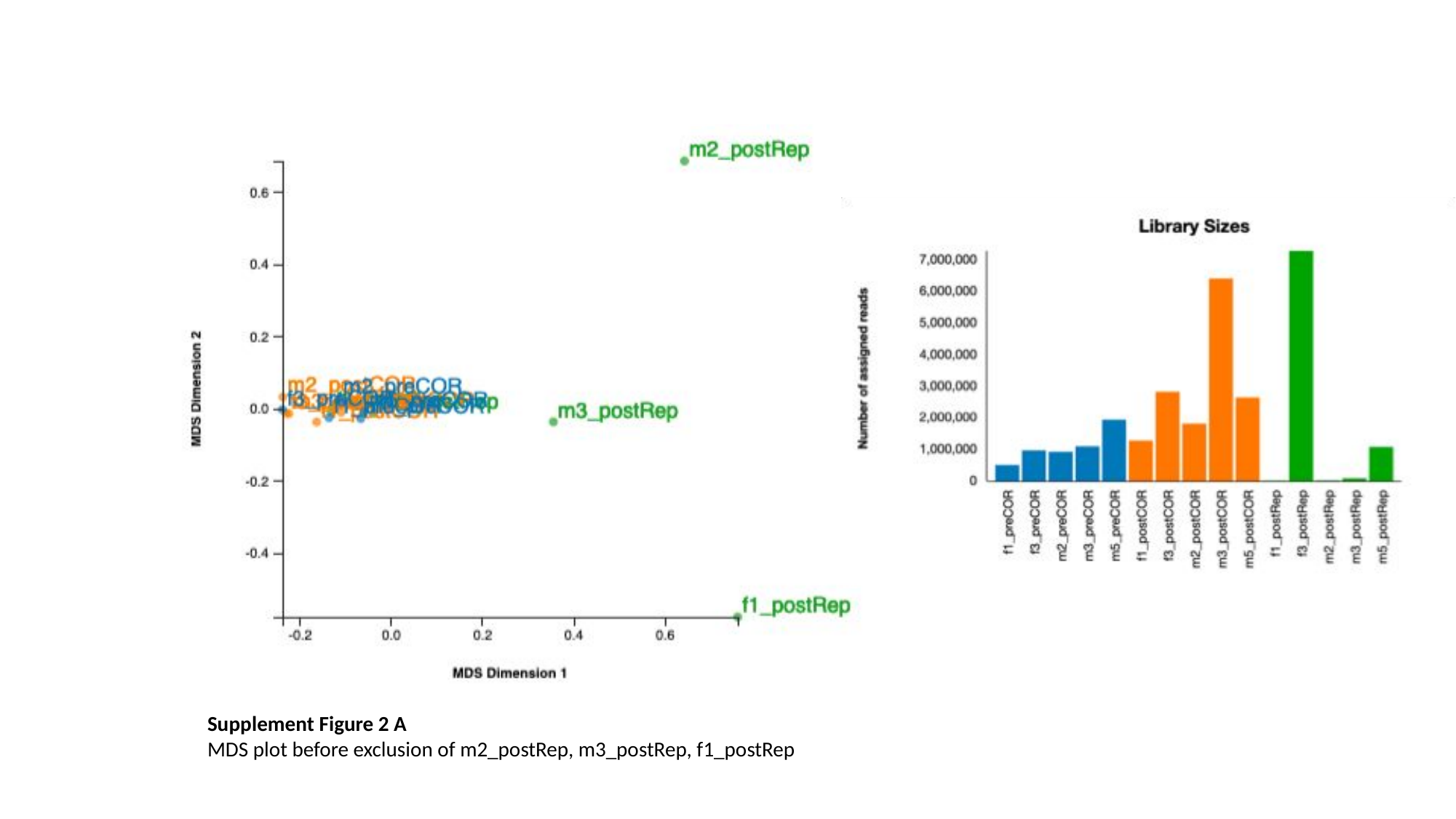

Supplement Figure 2 A
MDS plot before exclusion of m2_postRep, m3_postRep, f1_postRep

## Slide 2
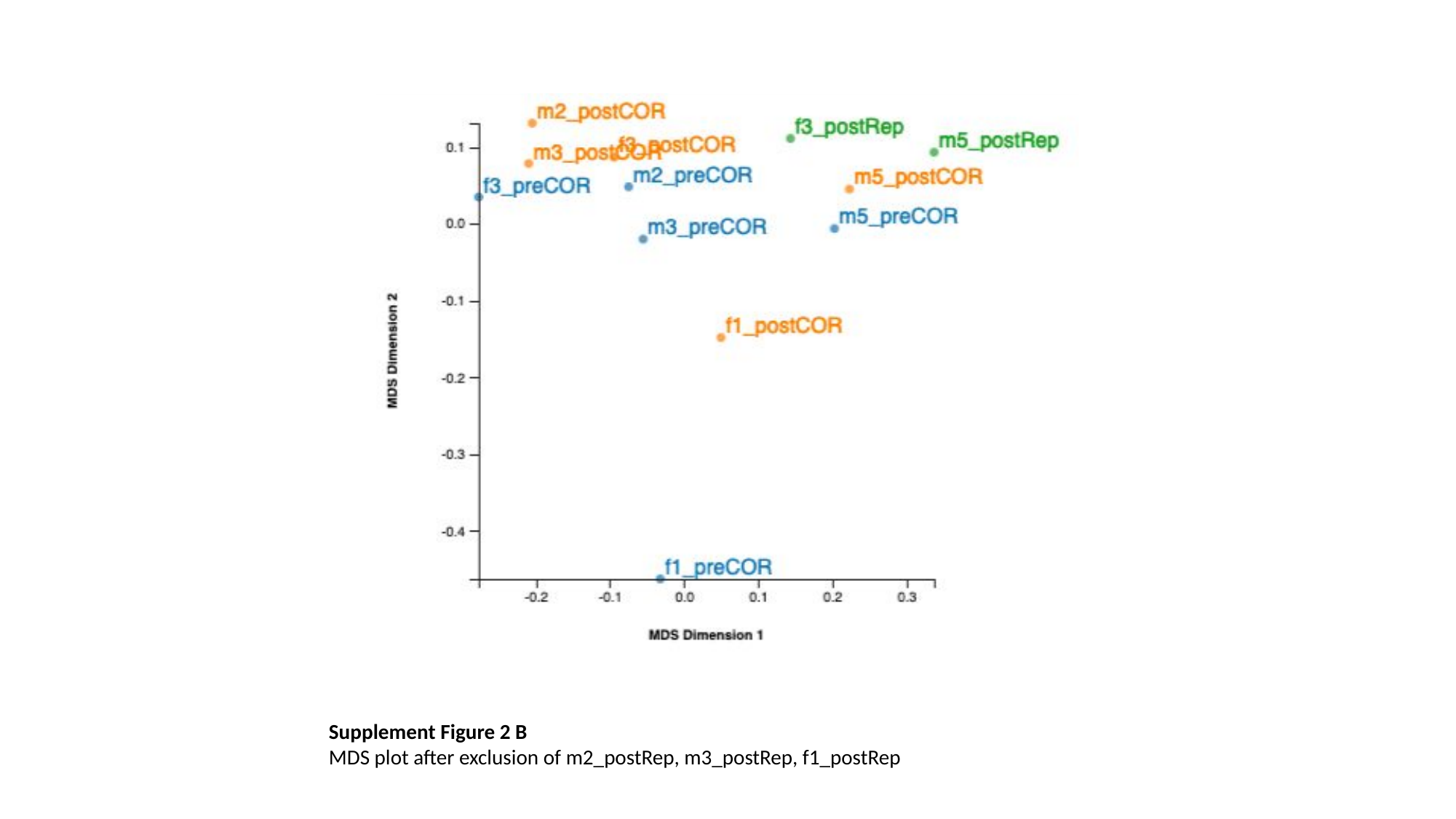

Supplement Figure 2 B
MDS plot after exclusion of m2_postRep, m3_postRep, f1_postRep

## Slide 3
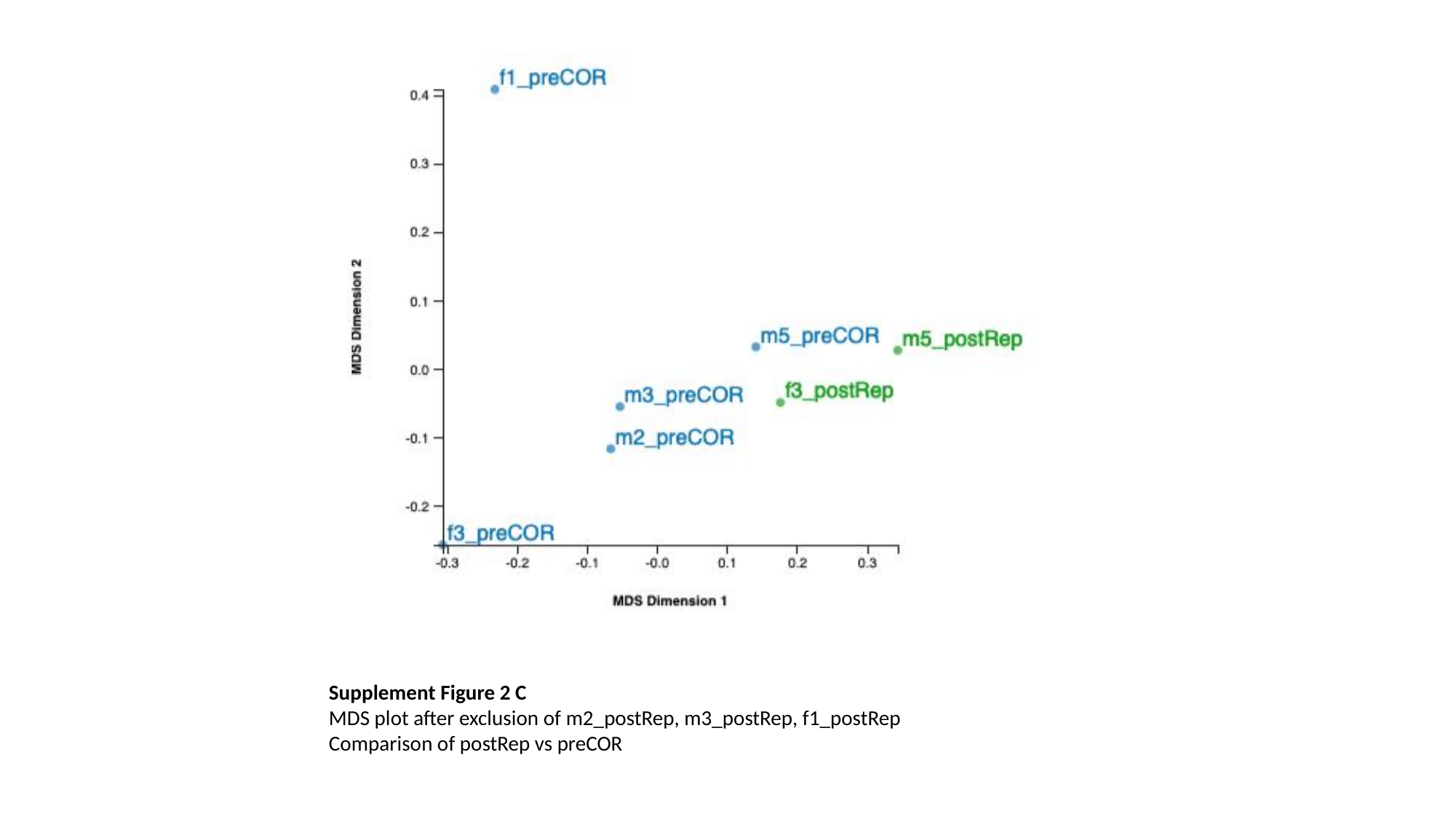

Supplement Figure 2 C
MDS plot after exclusion of m2_postRep, m3_postRep, f1_postRep
Comparison of postRep vs preCOR

## Slide 4
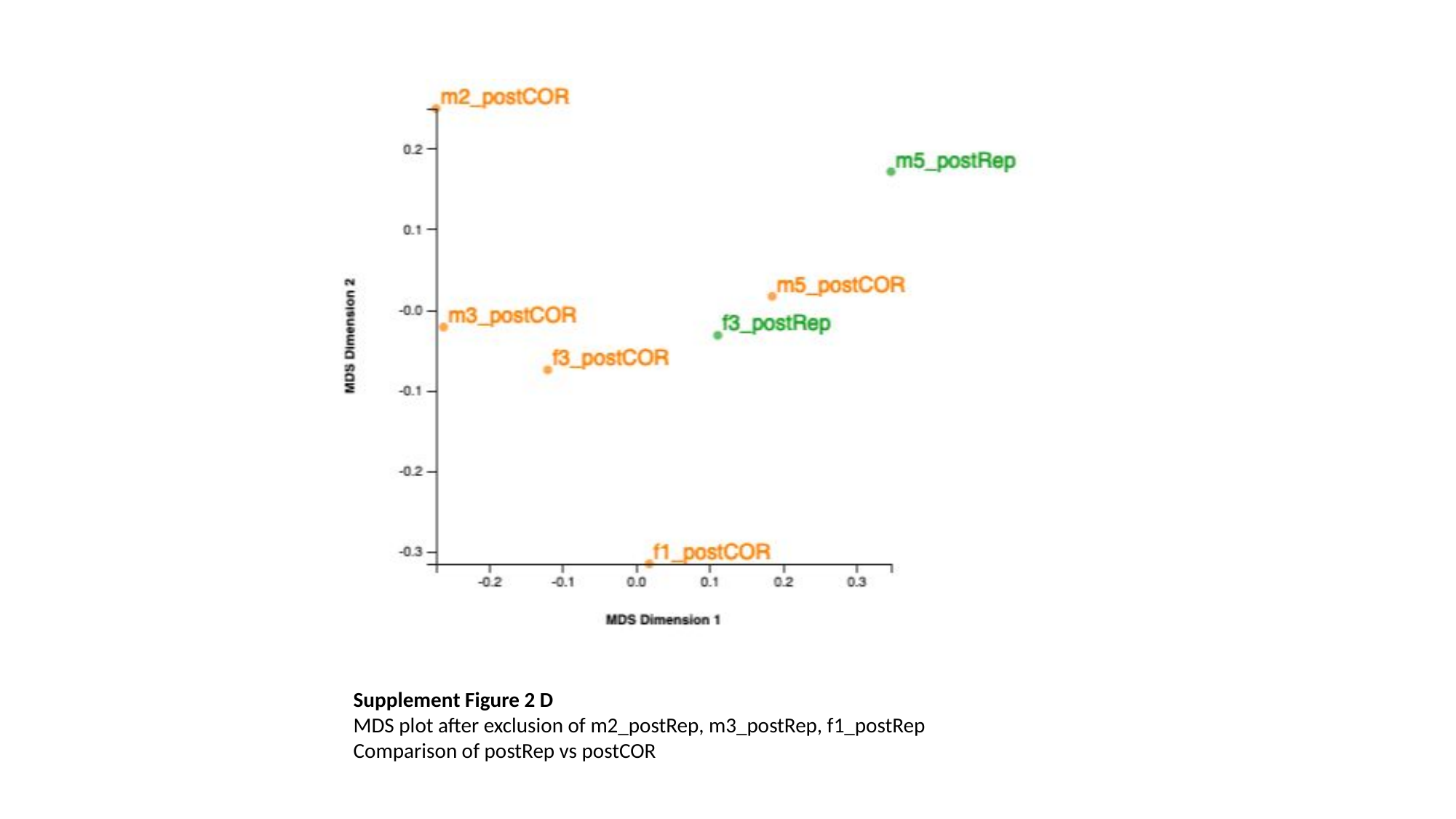

Supplement Figure 2 D
MDS plot after exclusion of m2_postRep, m3_postRep, f1_postRep
Comparison of postRep vs postCOR

## Slide 5
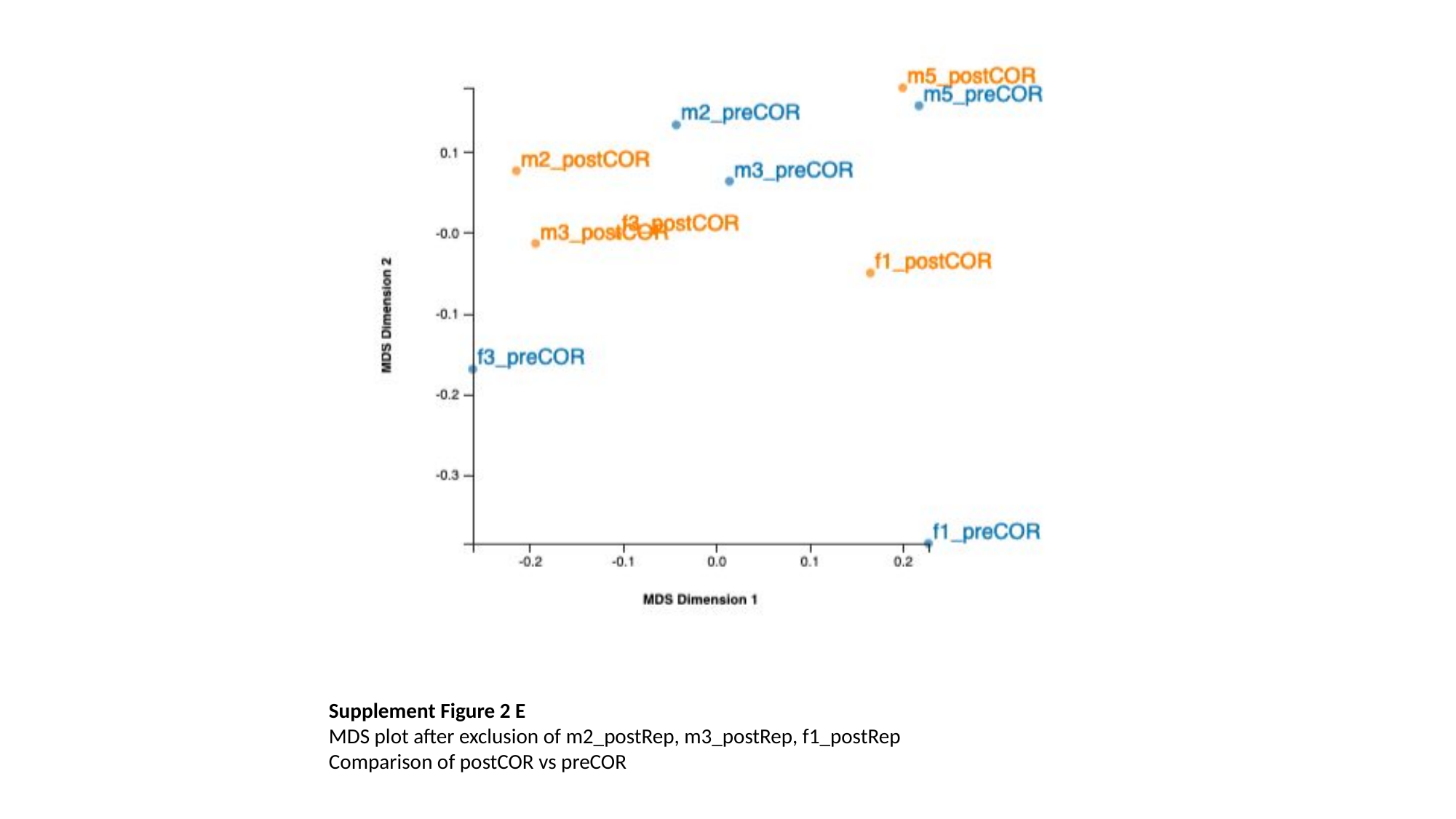

Supplement Figure 2 E
MDS plot after exclusion of m2_postRep, m3_postRep, f1_postRep
Comparison of postCOR vs preCOR
