# Supplementary material for: Transcriptomic profiles of human livers undergoing rewarming machine perfusion before transplantation—first insights
Source: Funct Integr Genomics. 2021 Mar 17;21(3-4):367–76. doi: 10.1007/s10142-021-00781-0 (PMC8298250; doi:10.1007/s10142-021-00781-0)
Supplement: Supplementary file 4 — (PPTX 1519 kb) [file 10142_2021_781_MOESM4_ESM.pptx]

## Slide 1
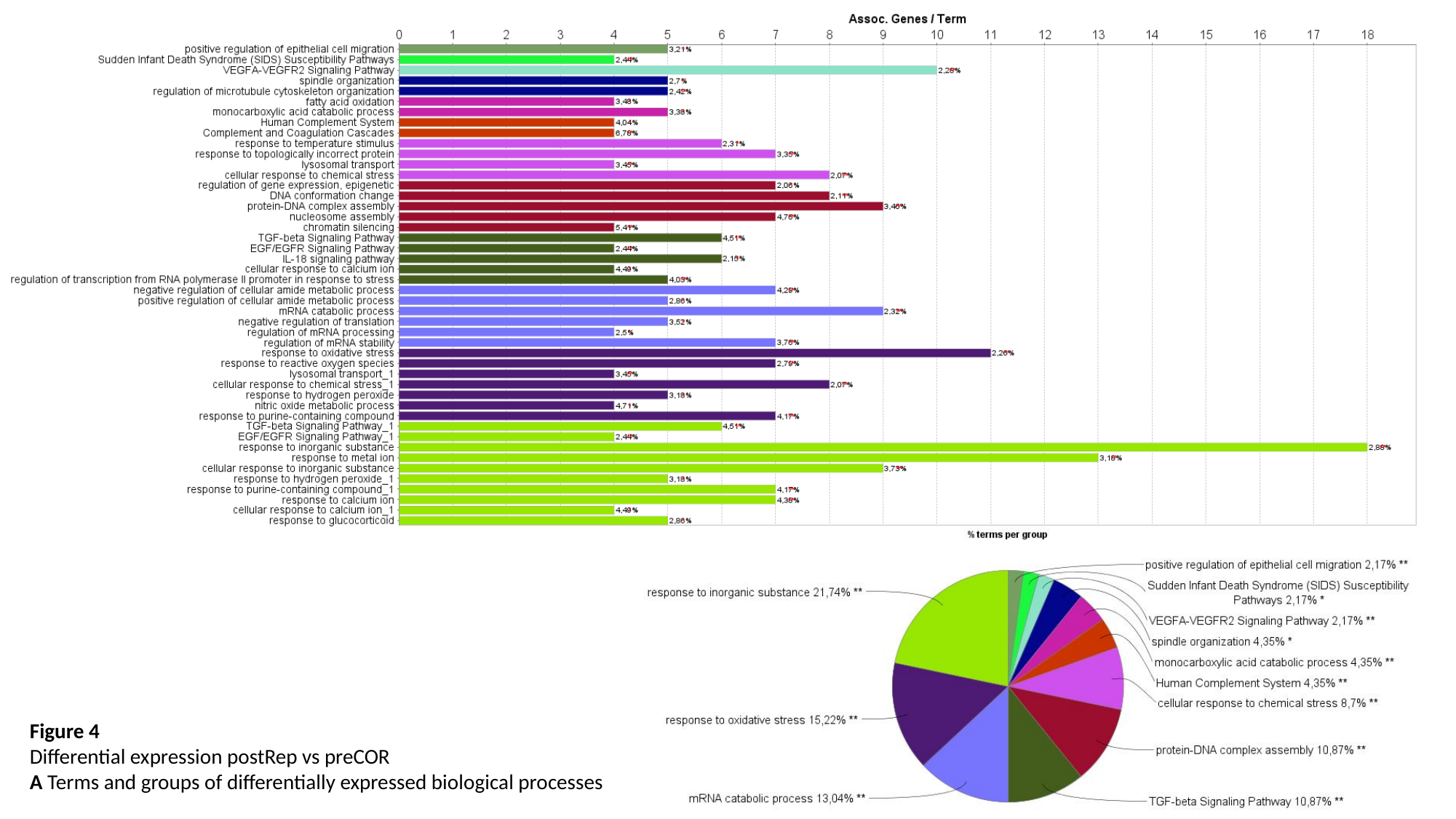

Figure 4
Differential expression postRep vs preCOR
A Terms and groups of differentially expressed biological processes

## Slide 2
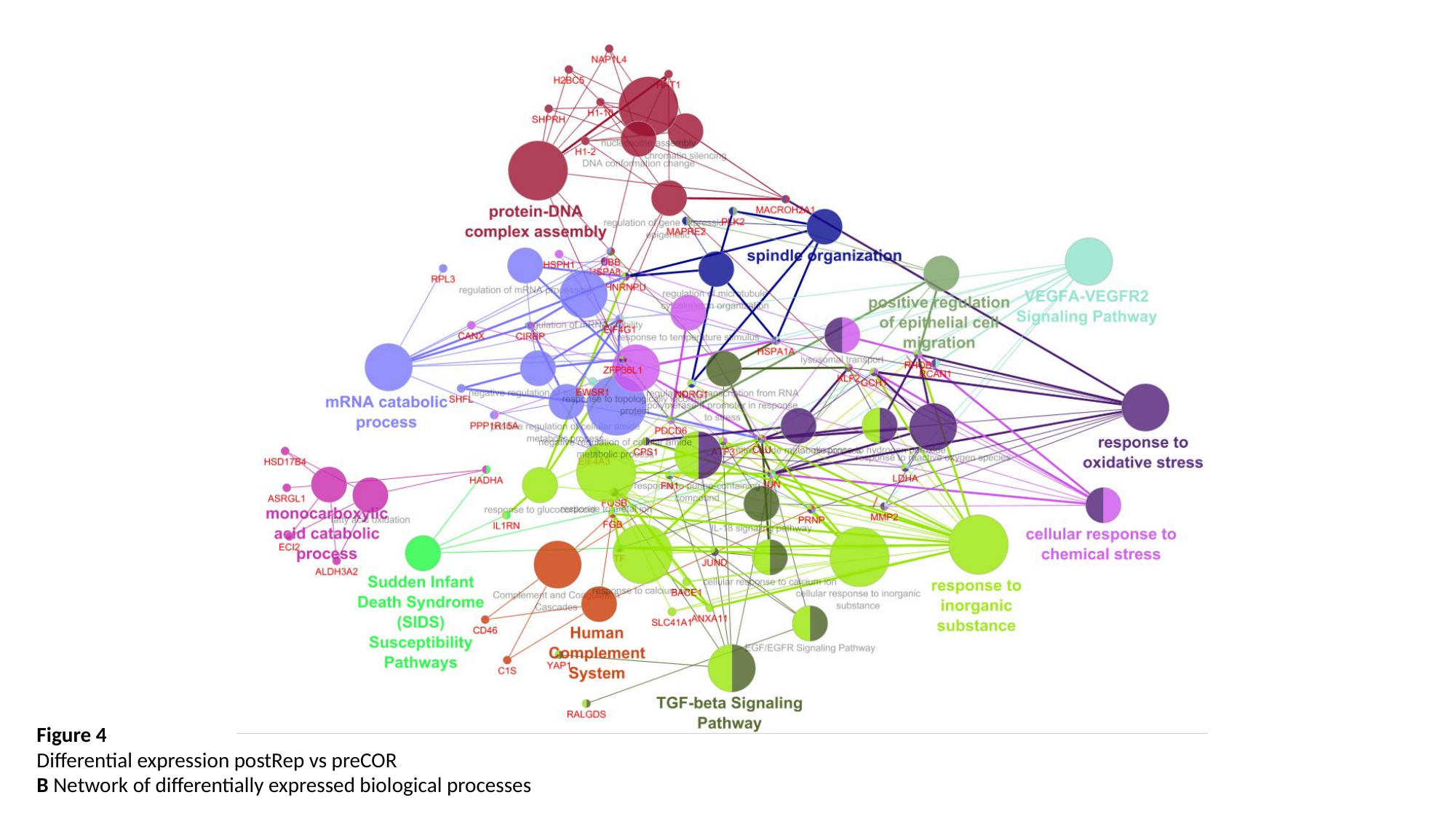

Figure 4
Differential expression postRep vs preCOR
B Network of differentially expressed biological processes

## Slide 3
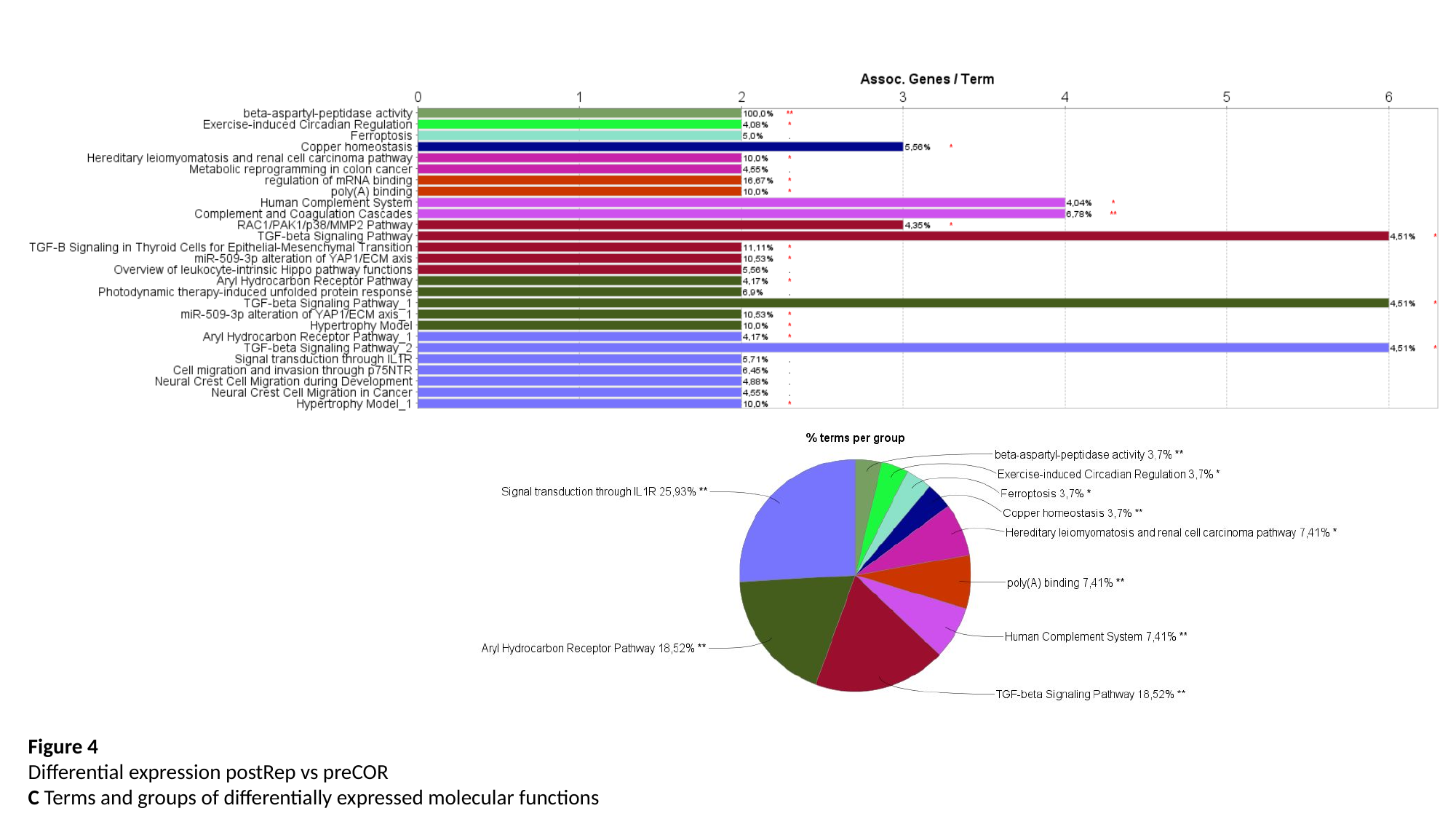

Figure 4
Differential expression postRep vs preCOR
C Terms and groups of differentially expressed molecular functions

## Slide 4
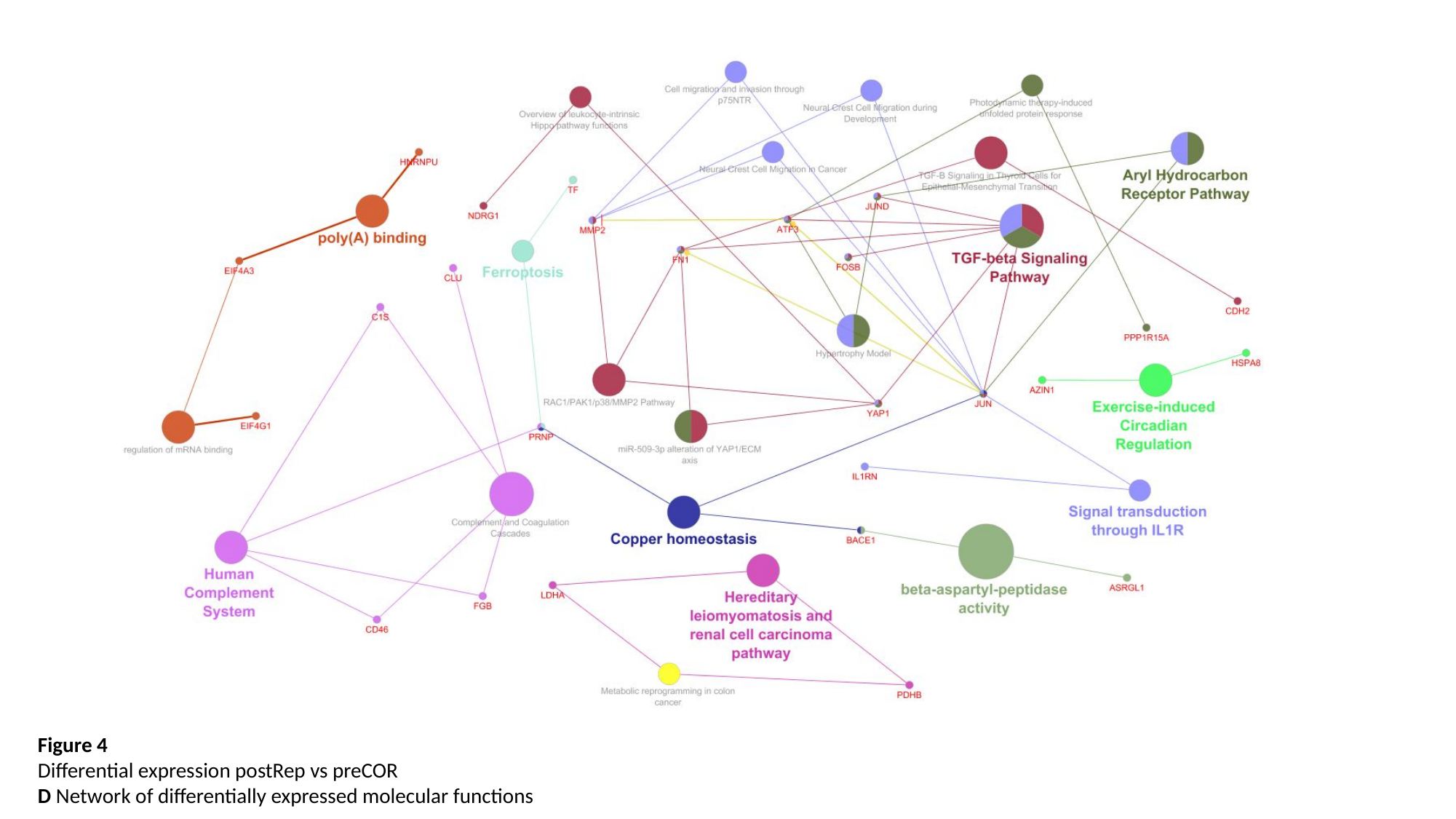

Figure 4
Differential expression postRep vs preCOR
D Network of differentially expressed molecular functions

## Slide 5
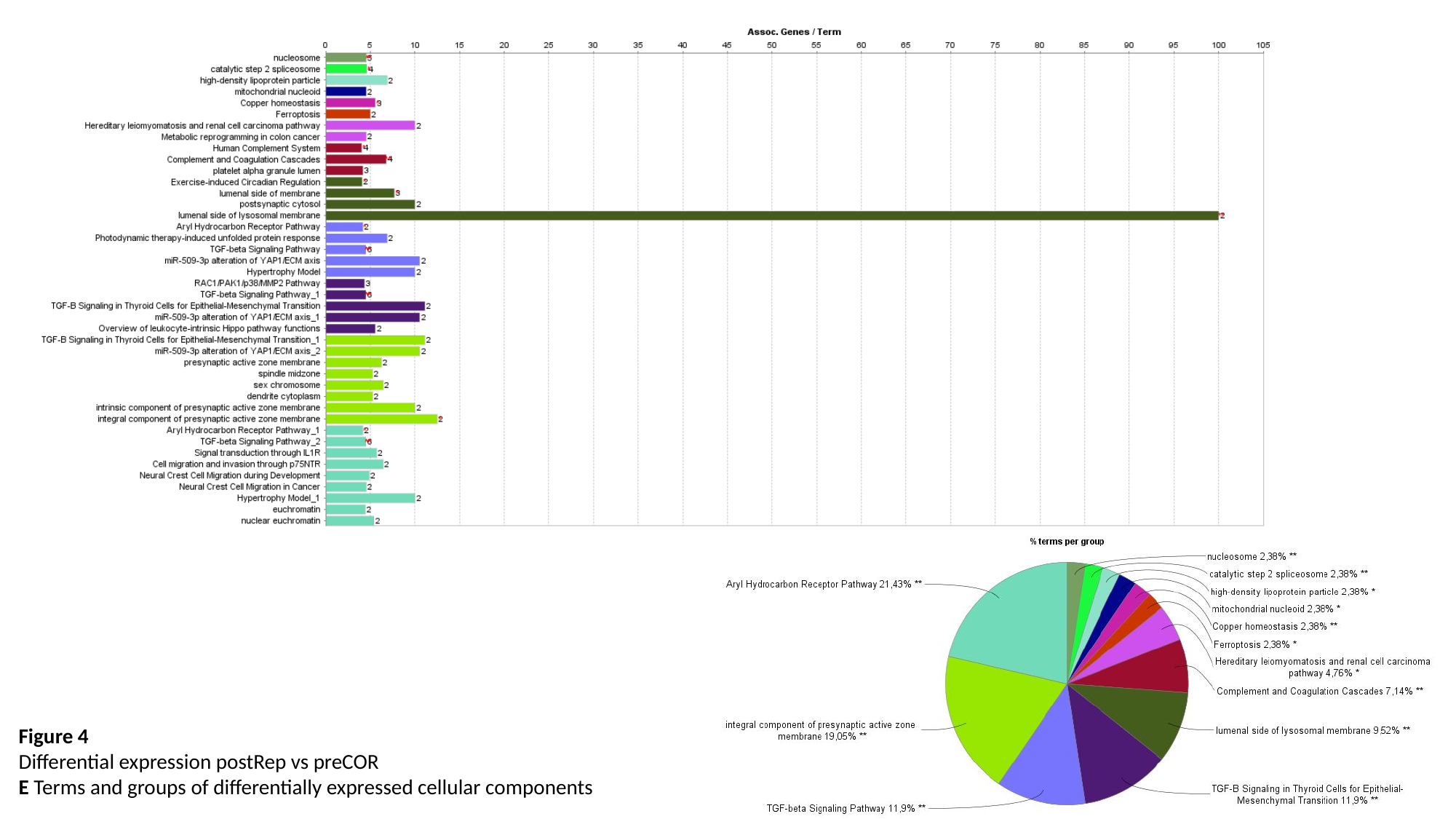

Figure 4
Differential expression postRep vs preCOR
E Terms and groups of differentially expressed cellular components

## Slide 6
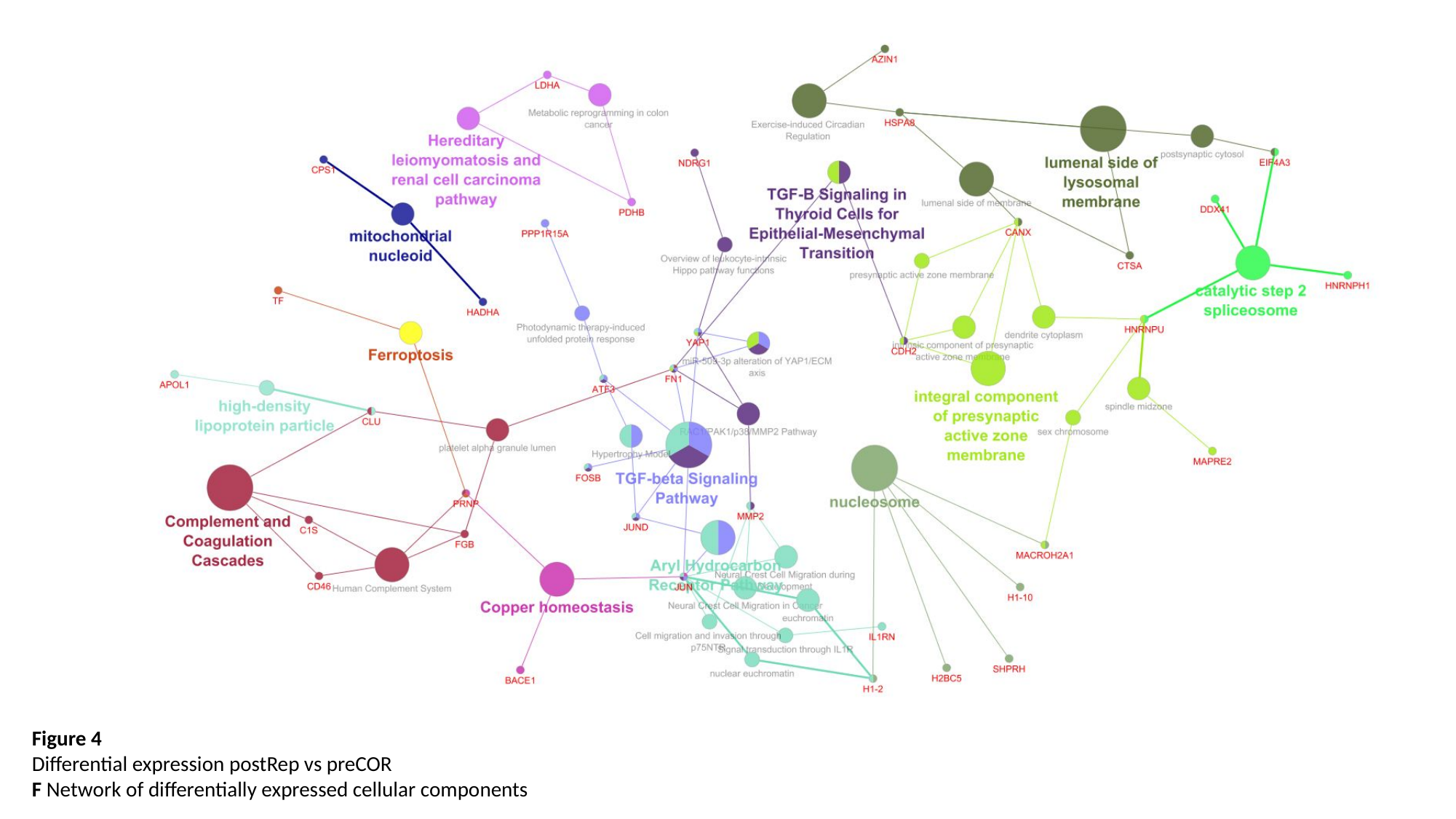

Figure 4
Differential expression postRep vs preCOR
F Network of differentially expressed cellular components
