# Supplementary material for: Transcriptomic profiles of human livers undergoing rewarming machine perfusion before transplantation—first insights
Source: Funct Integr Genomics. 2021 Mar 17;21(3-4):367–76. doi: 10.1007/s10142-021-00781-0 (PMC8298250; doi:10.1007/s10142-021-00781-0)
Supplement: Supplementary file 6 — (DOCX 13 kb) [file 10142_2021_781_MOESM6_ESM.docx]

**Supplementrary Table S2 Quality control; Number of reads**

| **preCOR** | **Number of reads** | **% Trimmed reads** | **postCOR** | **Number of reads** | **% Trimmed reads** | **postRep** | **Number of reads** | **% Trimmed reads** |
| --- | --- | --- | --- | --- | --- | --- | --- | --- |
| f1 | 768679 | 96,51 | f1 | 1716640 | 97,19 | f1 | 25417 | 95,22 |
| f3 | 1517210 | 96,75 | f3 | 3787860 | 96,83 | f3 | 8797710 | 98,41 |
| m2 | 1335700 | 97,43 | m2 | 2643850 | 96,82 | m2 | 41227 | 96,26 |
| m3 | 1526980 | 97,02 | m3 | 8943070 | 98,40 | m3 | 141306 | 96,90 |
| m5 | 2701220 | 96,71 | m5 | 3462320 | 97,07 | m5 | 1498330 | 96,74 |

Quality control of the raw reads revealed overall high sequencing quality. In all samples, more than 95% of all reads were eligible for further analysis after read trimming. (Table XY number of reads). Three samples showed very low library sizes (f1_postRep, m2_postRep, m3_postRep).
